# A rare and immediate onset of pneumocephalus following neuraxial needle insertion: A case report and review of the literature

**DOI:** 10.1097/MD.0000000000046537

**Published:** 2025-12-12

**Authors:** Ameer Shawar, Kenana Altell, Abdallah Najjar, Derar Zatari, Ahmad Abumunshar

**Affiliations:** a Department of Medicine, Hebron University, Hebron, Palestine; b Department of Anesthesiology, Al-Ahli Hospital, Hebron, Palestine.

**Keywords:** anesthesia, obstetric anesthesia, pneumocephalus

## Abstract

**Rationale:**

Pneumocephalus is a rare but potentially serious complication of neuraxial anesthesia, often misdiagnosed as post-dural puncture headache (PDPH) in obstetric settings. This case is unique for its immediate onset following a single epidural attempt using the loss-of-resistance to air (LORA) technique during labor. The report emphasizes the importance of vigilance, early imaging, and the potential advantage of using the loss-of-resistance to saline method to minimize the risk of intracranial air entry.

**Patient concerns:**

A 25-year-old woman in active labor developed sudden severe headache, neck pain, and upper back discomfort during the first epidural attempt. Despite reassurance and analgesics, symptoms persisted postpartum, causing significant distress and concern for neurological complications.

**Diagnoses:**

While PDPH was initially suspected, the headache’s persistence, partial non-postural nature, and poor analgesic response prompted further investigation. Non-contrast brain computed tomography revealed multiple intracranial gas locules in the lateral, third, and fourth ventricles and interpeduncular cistern, confirming post-dural puncture pneumocephalus.

**Interventions:**

An epidural blood patch provided rapid but short-lived relief, with symptoms recurring within 30 minutes. The patient was transferred to the surgical intensive care unit for conservative management, including high-flow oxygen therapy, supine positioning, intravenous fluids, analgesics, and high-caffeine beverages. Serial assessments and repeat computed tomography scans monitored air resolution. On postpartum day 8, she developed transient chest pain and shortness of breath; cardiopulmonary investigations were negative.

**Outcomes:**

The patient experienced gradual symptom improvement over 4 days, with a progressive reduction in headache intensity and no new neurological deficits. Follow-up imaging demonstrated near-complete resolution of pneumocephalus. She was discharged on postpartum day 10 with only mild, intermittent headaches, which fully resolved by outpatient follow-up, and she remained neurologically intact throughout, indicating a complete recovery.

**Lessons:**

Pneumocephalus should be considered in postpartum patients with acute or atypical headache after neuraxial anesthesia, particularly with LORA use. While symptoms can mimic PDPH, differences in onset and headache characteristics warrant early neuroimaging. Conservative measures, especially high-flow oxygen, are highly effective for non-tension pneumocephalus. Preference for the loss-of-resistance to saline technique over LORA may significantly reduce the risk of this complication, enhancing safety in obstetric anesthesia.

## 1. Introduction

Pneumocephalus, defined as the presence of air within the cranial cavity, is a rare but potentially serious complication of neuraxial anesthesia. Although it is more commonly associated with cranial trauma or neurosurgical procedures, instances following epidural anesthesia, particularly in obstetric settings, have been documented.^[1]^

The pathophysiology often involves inadvertent dural puncture during epidural placement, which allows air to enter the subarachnoid or subdural spaces. This risk increases when the loss-of-resistance to air (LORA) technique is employed. The air introduced during this process can ascend intracranially, leading to pneumocephalus. The use of air, as opposed to saline, to identify the epidural space has been implicated in several cases of pneumocephalus epidural anesthesia.^[2,3]^

Clinically, pneumocephalus often manifests with sudden-onset headaches, which may mimic post-dural puncture headache (PDPH). However, unlike PDPH, pneumocephalus-related headaches are typically non-postural and may be accompanied by additional neurological symptoms, such as nausea, vomiting, neck stiffness, or cranial nerve palsies.^[4]^ Delayed presentation, as seen in some cases, further complicates the diagnostic process.

Computed tomography (CT) remains the diagnostic modality of choice and can detect minimal volumes of intracranial air. Management typically involves conservative measures, such as bed rest, analgesia, hydration, and administration of high-flow oxygen to facilitate the resorption of intracranial air. Hyperbaric oxygen has been successfully employed in more severe or refractory cases.^[5]^

Although pneumocephalus following neuraxial anesthesia remains rare, recent case reports highlight its potential association with epidural procedures, particularly when air is used in the LOR technique.^[3]^ This underscores the importance of meticulous techniques, early recognition, and prompt management to mitigate adverse outcomes.

## 2. Case presentation

**Figure 1.**
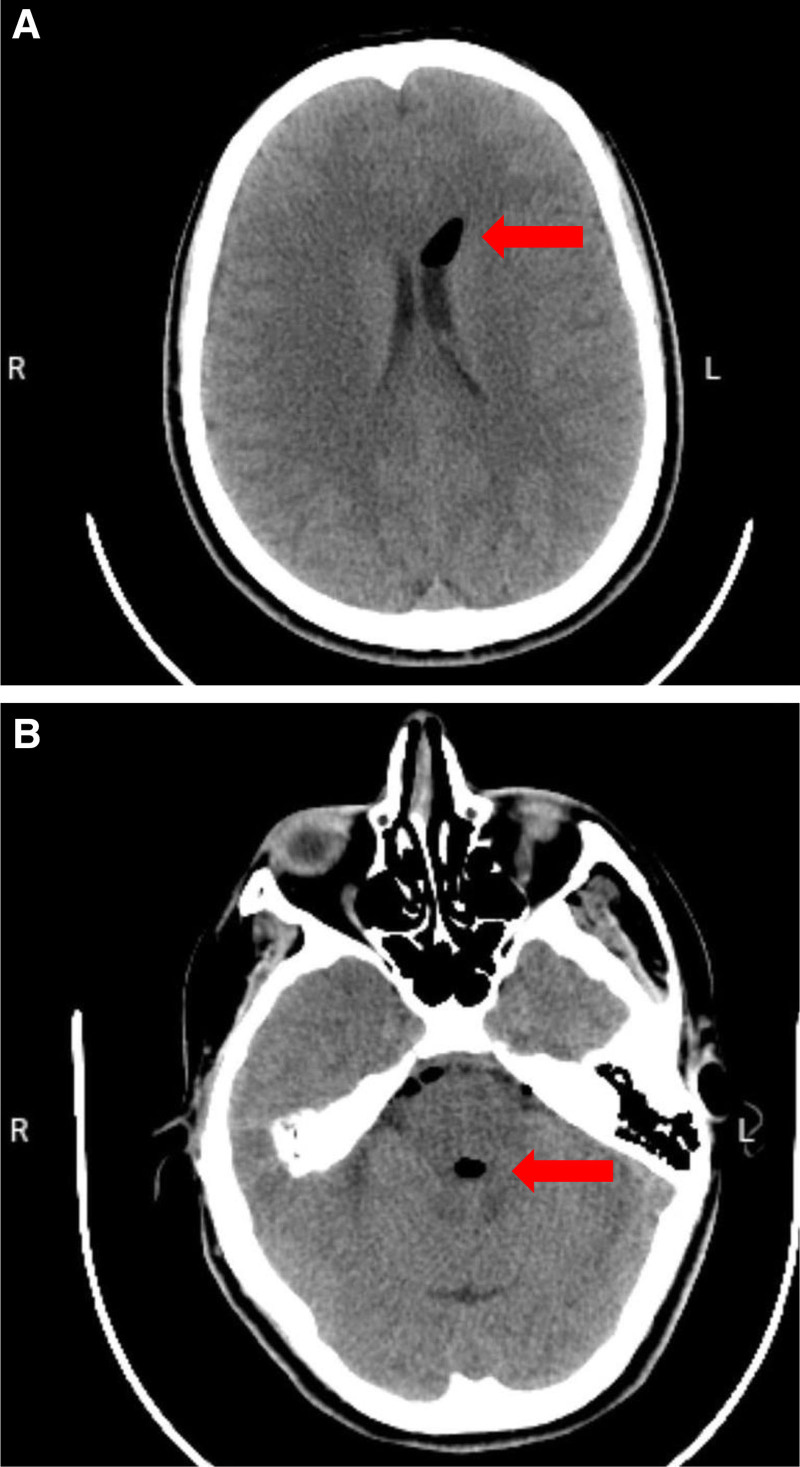
(A) Axial non-contrast CT scan of the brain showing a well-defined, crescent-shaped hypodense area (red arrow) consistent with intracranial air within the frontal horn of the left lateral ventricle, indicative of pneumocephalus. (B) Initial axial non-contrast CT scan of the brain showing pneumocephalus with air collection in the prepontine cistern (red arrow), consistent with peripontine pneumocephalus. CT = computed tomography.

**Figure 2.**
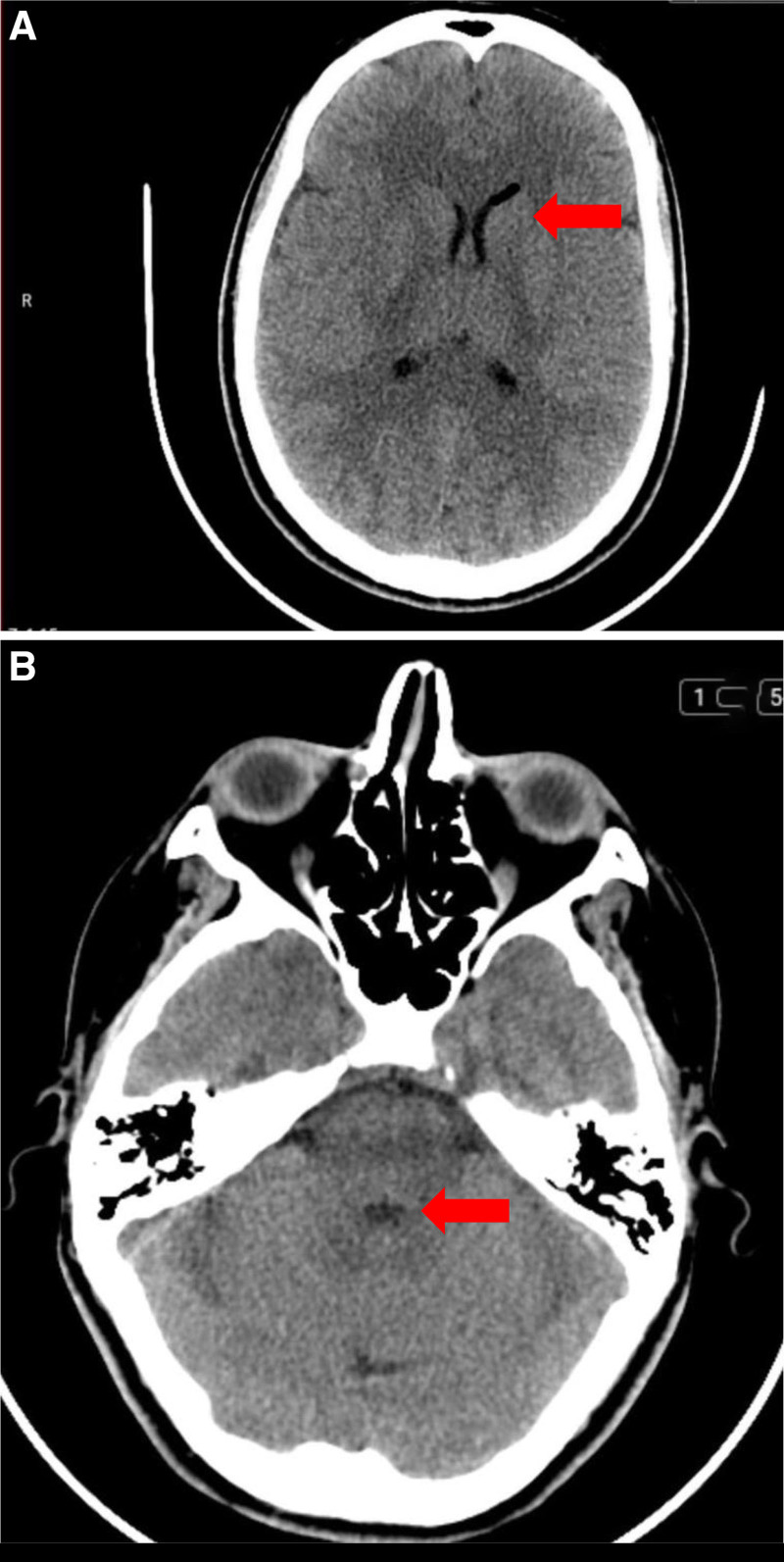
(A) Follow-up axial non-contrast CT scan of the brain demonstrating partial resolution of intracranial air (red arrow), with normalization of ventricular anatomy and with minimal residual pneumocephalus. (B) Follow-up axial non-contrast CT scan showing complete resolution of the previously seen peripontine pneumocephalus, with no residual intracranial air (red arrow). CT = computed tomography.

A 25-year-old female G2P0A1, at 36 weeks and 2 days of gestation, presented to the obstetrics department complaining of labor pain and was admitted to the hospital to proceed with vaginal delivery. Her obstetric history was significant for an ectopic pregnancy managed with salpingectomy. For labor pain management, the patient requested epidural anesthesia. Vital signs were normal and a complete blood count was unremarkable. The patient denied taking anticoagulants. A consent form was signed before starting the procedure with proper explanation for possible complications. A Tuohy needle was inserted at the L3-L4 interspace under sterile conditions using the LORA technique. Despite advancing the needle to a depth of 6 cm, there was no loss of resistance, and no cerebrospinal fluid or blood was aspirated. During this procedure, the patient began complaining of bi-frontal and bi-occipital headache accompanied by neck pain and upper back pain. Neurological examination showed intact power and sensation in both upper and lower limbs. The pain was gradual in onset, progressive in course and moderate in severity. Due to the patient’s anxiety, irritability, and arched back during labor, her pain was initially misinterpreted as a stress-induced muscle spasm. The Tuohy needle was withdrawn and reinserted at the L3-L4 interspace, with loss of resistance to air noted at 5.5 cm. A catheter was then successfully placed and fixed at 11 cm. Aspiration was negative for CSF or blood, and successful epidural placement was confirmed by a positive gravity test and pain relief after administering lidocaine. A loading dose of bupivacaine 0.125% (10 mL) was administered followed by a continuous infusion of the same concentration at a rate of 10 mL/h. The patient’s headache, neck, and upper back pain persisted throughout labor. However, the pain related to labor contractions responded well to epidural analgesia. Repeated evaluations of the patient’s pain were done by the medical team including an anesthesiologist, the pain was attributed to labor stress muscle spasm and stress-related headache. The patient gave birth via normal spontaneous vaginal delivery. Postpartum, the headache and back pain continued and she was reevaluated multiple times. Intravenous analgesics including nonsteroidal anti-inflammatory drugs and paracetamol were administered several times but with limited improvement in headache and neck pain. Notably, the pain worsened in the upright position and was relieved partially by lying flat. On postpartum day (PPD 1), the patient was reevaluated by a specialist. Despite the ongoing symptoms, the patient chose to leave and was discharged against medical advice with oral analgesics. On PDD 6, the patient presented to hospital with a severe bi-frontal and bi-occipital headache, associated with neck pain and dizziness. The headache was gradual in onset, progressive in course, aggravated by sitting in an upright position and standing, and partially relieved by analgesics and lying flat especially on her right side. The patient was evaluated by anesthesiology and obstetrics/gynecology specialists and a neurological examination showed a GCS of 15/15 and intact motor function and sensation in all 4 limbs. Examination of the epidural puncture site showed no signs of inflammation and laboratory investigations revealed no evidence of central nervous system infection or other abnormalities. The initial impression was PDPH. For that, an epidural blood patch was injected using 20 mL of autologous blood. The pain was relieved dramatically straight after the blood patch. However, symptoms started to recur 30 minutes later, and the headache was increasing in intensity again. The neurosurgery team was consulted and a non-contrast CT scan of the brain was ordered. Imaging revealed multiple gas locules (pneumocephalus) in the frontal horns of the lateral ventricles bilaterally as well as in the third, fourth ventricles and in the interpeduncular cistern as shown in Figure 1. Based on the imaging findings, a post-dural puncture pneumocephalus diagnosis was established. The patient was then transferred to the surgical intensive care unit for close observation and management as needed. The patient was kept in surgical intensive care unit for 2 days in which she showed clinical improvement with IV fluids, oxygen supplement via face mask, supine position, analgesics, and high-caffeine content beverages. A follow-up brain CT showed regression in gas locules (pneumocephalus) in the frontal horn of left lateral ventricle and complete resolution of gas locules in the frontal horn of right lateral, third and fourth ventricles, and in the interpeduncular cistern as shown in Figure 2. On PPD 8, the patient developed sudden intermittent parasternal chest pain with shortness of breath. Investigations including D-dimer, troponin, ECG, and chest CT angiography were all normal, ruling out pulmonary embolism and acute coronary syndrome. The chest pain resolved spontaneously. On PPD 9, she reported a gradual-onset, mild to moderate bi-frontal headache and neck pain, not posture-related and partially relieved by analgesics. A non-contrast brain CT showed near-complete resolution of the previously noted pneumocephalus, with no new findings. She was reassured and managed conservatively with analgesics and high-caffeine content beverages. On PPD 10, the patient was discharged in good clinical condition with improving symptoms and was prescribed oral analgesics and prophylactic antibiotics. At outpatient follow-up, she reported complete improvement of her symptoms with no recurrence of headache or other complaints.

## 3. Discussion

Pneumocephalus is a rare postoperative anesthetic complication, as the name indicated, it is characterized by the presence of air within the cranial vault. Although it is typically associated with cranial trauma, neurosurgical interventions, or infections involving the paranasal sinuses and skull base, its occurrence after general anesthesia for non-neurosurgical procedures remains exceedingly rare. This case underscores an important but often overlooked complication that may arise in the perioperative setting, even in patients without prior neurological pathology or cranial surgery.^[6]^

Several mechanisms have been proposed to explain pneumocephalus in the context of general anesthesia. One well-documented mechanism is the intraoperative use of nitrous oxide (NO), which diffuses into air-filled spaces more rapidly than nitrogen. If a dural defect or preexisting skull base defect is present (whether overt or occult), NO can expand the intracranial air, potentially exacerbating or precipitating the pneumocephalus. Positive pressure ventilation, especially with high peak airway pressures, may also contribute to increased intracranial air by forcing air into the intracranial cavity through microdefects in the skull base.^[7]^

In the context of regional anesthesia, particularly epidural anesthesia, the development of pneumocephalus is a rare but clinically significant complication. This is most commonly associated with the use of the LORA technique during the identification of the epidural space. When an inadvertent dural puncture occurs, air introduced into the epidural space may ascend cranially through the subarachnoid or subdural compartments, resulting in intracranial air accumulation. Several case reports have documented this occurrence in both obstetric and orthopedic surgical settings, highlighting the importance of technique selection and volume control during epidural placement.^[7,8]^

Headache is the most frequent symptom of pneumocephalus. It typically presents in the frontal or occipital regions and often worsens when the patient is upright, closely resembling PDPH, a more common cause of headache after spinal or epidural procedures. Both conditions can present with similar symptoms, including nausea, vomiting, neck pain or stiffness, visual disturbances, and cranial nerve deficits. Therefore, an accurate diagnosis relies on the clinical context, including the timeline of events and a high index of suspicion. Unlike PDPH, which usually develops gradually over 24 to 48 hours after a dural puncture, headaches due to pneumocephalus tend to have a sudden, severe onset. They may be aggravated not only by sitting or standing but also by general movement, and they often do not improve with lying down. Pneumocephalus can be classified based on the timing of either acute pneumocephalus, occurring within <72 hours of dural puncture or delayed pneumocephalus which occurs more than 72 hours following dural puncture.^[4,7]^

In our case, the patient started to have a frontal headache and neck rigidity immediately after a dural puncture procedure for labor pain relief, without evidence of poor performance or traumatic manipulation. Persistent headache (for 6 days) and confusion in the postoperative period prompted neuroimaging, which confirmed the presence of pneumocephalus. The absence of prior neurosurgical history, NO use, or sinus disease makes this case notable and raises the consideration of unrecognized anatomical defects or idiopathic processes as possible contributing factors.

CT imaging remains the gold standard for diagnosing pneumocephalus and allows differentiation between simple and tension pneumocephalus. In tension pneumocephalus, the “Mount Fuji sign” may be present, indicating increased intracranial pressure that requires emergent intervention. Fortunately, in our case, no radiologic signs of tension pneumocephalus were observed, just air-fluid level in the frontal area, so, conservative management in the Surgical-ICU with high-flow oxygen, complete rest and head elevation was effective.^[9]^

Because many studies have used the same management, good results have been observed. Li et al. (2024) reported a 52-year-old female who developed pneumocephalus following combined spinal-epidural anesthesia during hysteroscopic surgery, with postoperative severe headache and confirmed intracranial air. The patient was managed symptomatically and recovered without complications.^[10]^

Non-tension pneumocephalus is more common and generally has a favorable prognosis. It often resolves spontaneously with conservative management, including bed rest, head elevation, and high-flow oxygen therapy, to accelerate air resorption. Most patients recover without neurological deficits.^[11]^

From the anesthesiologist’s perspective, this case highlights the necessity of being vigilant about uncommon complications, especially when managing unexplained postoperative symptoms. Avoiding NO in patients with suspected or confirmed skull base defects, using careful ventilation strategies, use of saline (loss-of-resistance to saline) as a safer alternative to air, particularly in situations where dural puncture is suspected or the procedure is technically challenging, and promoting early imaging when neurological symptoms arise can aid timely diagnosis and treatment.

This case report is limited by its single-patient design, which restricts the ability to generalize the findings to broader populations. Additionally, long-term follow-up data were not available, preventing assessment of any delayed complications or recurrence. The diagnosis and management were based on clinical observation and imaging findings without comparative or control data. Despite these limitations, the report provides valuable clinical insight into an uncommon but significant complication of neuraxial anesthesia and underscores the importance of vigilance in similar clinical settings.

## 4. Conclusion

Pneumocephalus remains an uncommon but potentially serious complication of neuraxial anesthesia, particularly when the LORA technique is employed. This case highlights the diagnostic challenge in distinguishing PDPH from pneumocephalus in the postpartum setting, emphasizing the importance of clinical vigilance and early imaging when symptoms are atypical or persistent. Conservative management, including high-flow oxygen, bed rest, and analgesia, remains effective in most cases of non-tension pneumocephalus. Anesthesiologists should consider adopting the loss-of-resistance to saline technique to minimize the risk of intracranial air entry and improve patient safety. Greater awareness and early intervention can lead to favorable outcomes and prevent unnecessary morbidity in obstetric anesthesia.

## Author contributions

**Conceptualization:** Ameer Shawar, Kenana Altell, Abdallah Najjar, Ahmad Abumunshar.

**Data curation:** Ameer Shawar, Ahmad Abumunshar.

**Formal analysis:** Derar Zatari.

**Investigation:** Kenana Altell, Abdallah Najjar.

**Methodology:** Abdallah Najjar.

**Project administration:** Kenana Altell.

**Resources:** Kenana Altell, Abdallah Najjar, Derar Zatari.

**Software:** Derar Zatari.

**Supervision:** Derar Zatari, Ahmad Abumunshar.

**Validation:** Ameer Shawar, Derar Zatari, Ahmad Abumunshar.

**Visualization:** Ameer Shawar, Kenana Altell, Abdallah Najjar, Ahmad Abumunshar.

**Writing – original draft:** Kenana Altell.

**Writing – review & editing:** Ameer Shawar, Kenana Altell, Ahmad Abumunshar.
